# Mortality of working-age physicians compared to other high-skilled occupations in Austria from 1998 to 2020

**DOI:** 10.5271/sjweh.4169

**Published:** 2024-09-01

**Authors:** Claudia Zimmermann, Thomas Waldhoer, Eva Schernhammer, Susanne Strohmaier

**Affiliations:** 1 Department of Epidemiology, Center for Public Health, Medical University of Vienna, Vienna, Austria.; 2 Channing Division of Network Medicine, Department of Medicine, Brigham and Women’s Hospital and Harvard Medical School, Boston, MA, USA.; 3 Department of Epidemiology, Harvard T.H. Chan School of Public Health, Boston, MA, USA.

**Keywords:** cancer, cause-specific mortality, CVD, female, health profession, lifestyle, professional, registry study, standardized mortality ratio, standardized rate ratio, suicide

## Abstract

**Objectives:**

Physicians have been shown to have lower mortality compared to the general population, particularly regarding lifestyle-associated causes of death. Prior literature is divided on whether this is due to higher socioeconomic position (SEP), healthier lifestyle, or other specific occupational characteristics. This study analyzed the mortality of Austrian physicians compared to the general population and other (health) professionals with a similar SEP, and investigated patterns of lifestyle-associated mortality among physicians.

**Methods:**

Data from professional associations and cause-of-death statistics were collated to determine causes of death for all occupational groups. Gender-specific age-standardized mortality rates (ASMR) and standardized rate ratios (SRR) were calculated to compare main causes of death [cancer, cardiovascular disease (CVD), external causes] among physicians to other (health) professionals and the general population. Standardized mortality ratios (SMR) were calculated for more detailed causes of death in physicians compared to the general population.

**Results:**

Physicians had lower all-cause mortality than the general population [SRR 0.45, 95% confidence interval (CI) 0.41–0.49 for males and SRR 0.60, 95% CI 0.54–0.66 for females] and health professionals (SRR 0.72, 95% CI 0.60–0.88 for males and SRR 0.77, 95% CI 0.63–0.93 for females), mostly due to low CVD and cancer mortality. SMR for detailed causes of death among physicians exhibited a pattern of particularly low mortality in lifestyle-associated causes of death and an increased SMR for suicide among female physicians (SMR 1.58, 95% CI 1.22–2.02).

**Conclusions:**

This study confirmed lower mortality among physicians compared to the general population and compared to other (health) professionals. Low physician mortality can be primarily explained by lifestyle-associated causes of death.

All occupations are characterized by work-related health risks that can affect mortality. Differences in mortality across various occupations are difficult to examine since there are many factors to consider when making comparisons between occupational groups. One important influence on mortality that is indirectly linked to occupation is socioeconomic position (SEP). Across countries and health systems, there is consistent evidence for an association between SEP and mortality, indicating that those with lower SEP die younger. This holds true for all-cause mortality (1, 2) as well as mortality from various cancers (3, 4), cardiovascular disease (CVD) (5, 6) and suicide (7, 8), among other specific causes of death. There is a strong connection between occupation and SEP: occupational category is often used as an indicator for SEP, in addition to other variables like income and educational level. All of these measures have repeatedly been shown to have a similar, inverse relationship with mortality (6). In order to serve as a suitable criterion for SEP, occupation needs to be captured in rather broad classification systems. One frequently used example would be the International Standard Classification of Occupations (ISCO), where the hierarchy among ten broad occupational categories more closely reflects educational levels (9). However, a broad categorization does not allow for a comparison of individual occupations and thus obscures differences in work-related risk factors for mortality among professions with a similar SEP. The examination of these differences is not only important to identify causal pathways of occupational health hazards, but also to develop targeted prevention measures that are based on occupation-specific risk profiles.

Physicians are one of the more well-researched occupational groups regarding mortality, with several studies reporting lower mortality and longer life expectancy compared to the general population (10–15). The exception is suicide (16, 17), especially among female physicians. Given that physicians have a relatively high SEP, a lower mortality than the general population is to be expected. Health behaviors like smoking, alcohol consumption, physical inactivity, and unhealthy dietary patterns appear to be on the causal pathways between SEP and mortality, as has previously been shown (18). Physicians tend to smoke considerably less than the general population, and even less than some other health professionals (19). They also exercise more (20), are less overweight and obese (21) and tend to eat more fruit and vegetables (22) compared to the general population. Alcohol use appears to be a notable exception. Older studies showed similar consumption levels compared to the general population, whereas a recent review found an increase in problematic alcohol use among physicians that could mean higher levels in some physician populations (23). Physicians are also affected by some specific occupational risk factors that have been shown to increase mortality. For example, shift work has been repeatedly reported to be associated with higher all-cause (24), CVD (25) and cancer mortality (26, 27). A review on workplace-related risk factors (28) also found evidence that overtime and job stress [particularly low job control (29)] can increase CVD mortality, similar to a meta-analysis that reported the same for long working hours (30).

A particularly relevant characteristic of physicians as an occupational group is their extensive knowledge of health promotion and disease prevention, which might contribute to a healthier lifestyle and lower mortality rates. However, this association cannot be assessed by comparing physicians to the general population, due to confounding by SEP. Another potential source of bias is the healthy worker effect: the general population can be expected to have higher mortality rates since it includes individuals that are unable to work due to ill health (31). Physicians are classified as professionals in the second highest category of the ISCO, so a comparison to other professionals with similar SEP that are equally affected by the healthy worker effect would help circumvent this potential bias. Few studies have compared physician mortality to other occupational groups on a similar socioeconomic or educational level. The results are conflicting: two studies reported lower mortality of physicians (11, 12), another study concluded it was comparable to other occupational groups with similar SEP (10), and yet another found it to be higher (15). None of these studies compared physicians to other health professionals, which would be another interesting comparison group due to their high levels of health-related knowledge.

This study has two aims: to compare mortality rates from (i) the main causes of death among physicians to a selection of high-skilled health professionals (dentists, veterinarians, pharmacists), high-skilled professionals in general (health professions and notaries, lawyers, tax advisors/public accountants), as well as the general population; and (ii) a wider range of specific causes of death among physicians to the general population to identify a potential pattern of lifestyle-associated causes of death that might contribute to the lower all-cause mortality among physicians.

## Methods

### Study population and data collection

The study population included high-skilled occupational groups with established professional chambers in Austria. Membership in the respective chambers is mandatory in order to practice these professions in Austria, and most institutions agreed to provide data for this study: the Austrian Medical Chamber (physicians), the Dental Chamber (dentists), the Chamber of Veterinary Surgeons (veterinarians), the Chamber of Pharmacists, the Chamber of Tax Advisors and Public Accountants, the Chamber of Civil-Law Notaries, and the Austrian Bar (lawyers). The Chamber of Architects and Engineer Consultants declined the invitation to contribute, and the Chamber of Patent Attorneys was not included due to their small membership size (80 members in 2021).

The participating institutions provided lists of their deceased members to be used for data collation with the Austrian cause-of-death statistics, which is provided by Statistics Austria (the federal statistical office). Based on medically documented information on death certificates, it is generally considered one of the most reliable sources of health data in Austria (32). Additional information on yearly numbers of members and their age distribution (grouped by gender) was also provided by the professional chambers. Data on causes of death for the Austrian general population was obtained from Statistics Austria. Detailed information on the process of data collection is published elsewhere (33). The Ethics Committee of the Medical University of Vienna approved this research (protocol number 1372/2019).

### Cause of death categories

We categorized causes of death according to the International Classification of Diseases (ICD) version used at the time: ICD-9 up to 2001 and ICD-10 thereafter. The time periods of available data varied between chambers, and there were some deceased persons from each professional group whose cause of death could not be determined (eg, due to misspellings or incomplete names, name changes because of marriage, or dying abroad). The exact proportion of cases with missing causes of death could not be calculated for physicians and dentists because we used lists of former members rather than deceased members of the respective professional chambers. The proportion of cases with missing causes in the other occupational groups was 5.5–10.9%.

Mortality was grouped into main causes of death for the working-age population, with the following categories: cancer (ICD-10/ICD-9 codes C00–C97/1400–2089), CVD (I00–I00/3900–4599), external causes of death (V01–Y89/E8000–E9990), and all other causes of death. Suicide as a subcategory (X60–X84) of external causes of death was also included. For a more detailed analysis of physician mortality, a broader range of causes of death were selected based on statistical considerations. For the interpretation of these results, we specifically focused on lifestyle-associated causes of death, ie, cause-specific mortality with well-documented links to individual behaviors such as diet, physical activity, smoking, and substance abuse. This includes deaths from certain cardiovascular and metabolic diseases (eg, myocardial infarction or diabetes), as well as certain malignant neoplasms (eg, lung cancer, breast cancer), respiratory diseases (eg, chronic lower respiratory disease), and digestive diseases (eg, chronic liver disease).

### Statistical analysis

We calculated age-standardized mortality rates (ASMR) per 100 000 person-years for the main causes of death among physicians and several reference groups: health professionals (dentists, veterinarians, pharmacists), all professionals combined (health professionals and notaries, lawyers, tax advisors/public accountants) and the Austrian general population (which also includes the aforementioned occupational groups). Occupational groups were combined in order to reach the recommended minimum number of ten observed cases for each calculated ASMR (34). Rates were grouped by gender and calculated for the longest possible time period for which data was available from all professional groups (1998–2020). To ensure international comparability, the 2013 European Standard Population (35) was used for standardization based on 10-year age categories. The lower limit of the age range varied between professional groups and was typically not specified in the membership statistics, but can be assumed to be 21–25 years depending on the educational requirements of the profession. Since data quality did not permit to establish which deceased physicians were still professionally active and counted towards the chamber membership numbers after retirement, we chose to use an age cut-off. The upper limit of the age range was 65 years for pharmacists and 64 years for all other groups, which corresponds with the typical Austrian retirement age of 65 years. For the general population, we used a comparable age range of 21–64 years.

To compare mortality from main causes of death between physicians and different reference groups (health professionals, all professionals, general population), age-adjusted standardized rate ratios (SRR) were calculated. This outcome measure allows for comparisons of age-standardized rates across different populations and reference groups that are standardized to the same age distribution (36). We report 95% confidence intervals (CI) based on the Rothman & Greenland method (37).

To examine more specific causes of death among male and female physicians compared to the general population, we calculated standardized mortality ratios (SMR) with 95% CI using Fisher’s exact test (38). We chose SMR for this analysis because only one population and reference group are used and some case numbers for the more detailed causes of death were quite low (<20), in which case SMR are preferable to SRR (36). Indirect age-standardization was based on the Austrian general population as a reference group, again with 10-year age categories. Causes of death were selected to allow for the detection of a significant SMR of 0.5 among male physicians with 80% power and 5% type-I error (37), which mandated an expected number of deaths of 23. Exceptions were made for accidental poisonings and deaths of undetermined intent, which had lower expected numbers, but were of particular interest in order to rule out undetected suicides. To further increase power for this comparison, we used data from the maximum observation period for physicians (1986–2020).

All data analyses were performed with Stata, version 17 (StataCorp, College Station, TX, USA). The width of CI was not adjusted for multiplicity due to the explorative nature of the study.

## Results

### Mortality distribution for main causes of death

The cause of death could be identified for 1213 physicians who died over the span of 23 years (1998–2020) under the age of 65 years (see table 1). Across all groups, males roughly made up two thirds of all deaths. There was a very low number of deaths among female notaries (one case) and lawyers (four cases), so those two groups were excluded. As a result, it was not possible to use the same combination of other professionals in comparison with female physicians.

**Table 1:** Characteristics of study population groups for main causes of death ^a^ (1998–2020).

		Person-years	Deaths (known cause)		Cancer		CVD ^b^		External causes		Suicide ^c^		All other causes
			N		N (%)		N (%)		N (%)		N		N (%)
Males													
	Physicians	460 670	858		287 (33.4)		188 (21.9)		195 (22.7)		98		188 (21.9)
	Health professionals ^d^	111 897	279		89 (31.9)		71 (25.4)		60 (21.5)		36		59 (21.1)
	All professionals ^e^	279 525	574		205 (35.7)		145 (25.3)		125 (21.8)		65		99 (17.2)
	General population ^f^	58 209 357	196 899		63 960 (32.5)		45 131 (22.9)		33 763 (17.1)		14 500		54 045 (27.4)
Females													
	Physicians	366 433	355		188 (53.0)		32 (9.0)		72 (20.3)		43		63 (17.7)
	Health professionals ^d^	162 098	173		89 (51.4)		20 (11.6)		36 (20.8)		20		28 (16.2)
	All professionals ^e^	N/A	N/A		N/A		N/A		N/A		N/A		N/A
	General population ^f^	58 221 629	100 460		50 051 (49.8)		16 334 (16.3)		9 831 (9.8)		4 330		24244 (24.1)
Overall													
	Physicians	827 103	1 213		475 (39.2)		220 (18.1)		267 (22.0)		141		251 (20.7)
	Health professionals ^d^	273 995	452		178 (39.4)		91 (20.1)		96 (21.2)		56		87 (19.2)
	All professionals ^e^	N/A	N/A		N/A		N/A		N/A		N/A		N/A
	General population ^f^	116 430 986	297 359		114 011 (38.3)		61 465 (20.7)		43 594 (14.7)		18 830		78 289 (26.3)

^a^ ICD-10/ICD-9 codes: cancer C00-C97/1400-2089, CVD I00-I00/3900-4599, external causes V01-Y89/E8000-E9990, suicide X60-X84, Y87.0/E9500-E9590. ^b^ CVD = cardiovascular disease. ^c^ Suicide is a subcategory of external causes of death and thus not included in the percentage distribution. ^d^ Health professionals include dentists, veterinarians, and pharmacists. ^e^ All professionals include health professionals + notaries, lawyers, tax advisors and public accountants. ^f^ The general population also includes all aforementioned occupational groups.

The overall proportions of the main causes of death were fairly similar across all groups for cancer (close to 40%) and CVD (around 20%), whereas the physicians and health professionals had a higher percentage of deaths from external causes and a lower one for the remaining causes of death. For males, about a third of all deaths were due to cancer, compared with about 50% for females. Contrastingly, the fraction of CVD deaths was lower among women than men, particularly in physicians and health professionals. The proportion of deaths from external causes were roughly twice as high in female physicians and health professionals compared to women in the general population. A similar but weaker pattern can be seen among men. In all professional groups, more than half of the deaths from external causes were due to suicide.

### Main causes of death for physicians in comparison to different reference groups

Table 2 shows the ASMR for all-cause mortality and main causes of death among physicians and comparison groups, as well as the resulting SRR. For male physicians, the SRR in reference to health professionals were all <1 and thus indicated lower mortality, ranging from 0.61 (95% CI 0.41–0.91) for CVD and 0.82 (95% CI 0.55–1.22) for external causes. When male physicians were compared to all professionals, the SRR were mostly <1, ranging from 0.72 (95% CI 0.48–1.06) for CVD and 1.04 (95% CI 0.68–1.58) for other causes. When the general population was used as a reference group, the SRR were all significantly <1, ranging from 0.35 (95% CI 0.29–0.43) for other causes and 0.77 (95% CI 0.59–1.00) for suicide. With the exception of suicide, the SRR of male physicians in reference to health professionals or other professionals were higher than the respective SRR in reference to the general population.

**Table 2:** Age-standardized mortality rates (ASMR) per 100 000 person-years and standardized rate ratios (SRR) with 95% confidence intervals (CI) for physicians and comparison groups (1998–2020).

			Physicians		Health professionals ^a^		All professionals ^b^		General population ^c^		Physicians/ health professionals		Physicians / all professionals		Physicians / general population
			ASMR		ASMR		ASMR		ASMR		SRR (95% CI)		SRR (95% CI)		SRR (95% CI)
Males															
	All-cause mortality		158.4		218.6		188.9		354.3		0.72 (0.60–0.88) ^d^		0.84 (0.69–1.02)		0.45 (0.41–0.49) ^d^
	Cancer		51.9		69.5		67.1		116.7		0.75 (0.53–1.05)		0.77 (0.55–1.09)		0.44 (0.38–0.52) ^d^
	CVD ^e^		34.0		55.7		47.4		82.0		0.61 (0.41–0.91) ^d^		0.72 (0.48–1.06)		0.42 (0.34–0.51) ^d^
	External causes		38.3		46.8		41.4		58.5		0.82 (0.55–1.22)		0.93 (0.62–1.38)		0.65 (0.54–0.79) ^d^
		Suicide	19.2		27.8		21.3		25.1		0.69 (0.41–1.17)		0.90 (0.54–1.52)		0.77 (0.59–1.00) ^d^
	All other causes		34.1		46.6		32.9		97.1		0.73 (0.48–1.12)		1.04 (0.68–1.58)		0.35 (0.29–0.43) ^d^
Females															
	All-cause mortality		113.6		147.6		N/A		189.8		0.77 (0.63–0.93) ^d^		N/A		0.60 (0.54–0.66) ^d^
	Cancer		59.9		76.3		N/A		87.9		0.78 (0.56–1.11)		N/A		0.68 (0.58–0.80) ^d^
	CVD ^e^		10.4		16.5		N/A		28.7		0.63 (0.42–0.93) ^d^		N/A		0.36 (0.29–0.44) ^d^
	External causes		21.2		27.8		N/A		17.0		0.76 (0.51–1.14)		N/A		1.25 (1.03–1.52) ^d^
		Suicide	12.2		14.4		N/A		7.4		0.84 (0.50–1.42)		N/A		1.63 (1.25–2.14) ^d^
	All other causes		22.1		27.0		N/A		42.5		0.82 (0.54–1.25)		N/A		0.52 (0.42–0.64) ^d^

^a^ Health professionals include dentists, veterinarians, and pharmacists. ^b^ All professionals include health professionals + notaries, lawyers, tax advisors and public accountants. ^c^ The general population also includes all aforementioned occupational groups. ^d^ Statistically significant at 5%. ^e^ CVD = cardiovascular disease.

For female physicians, the SRR in reference to health professionals were all <1, with significant results for all-cause mortality (0.77; 95% CI 0.63–0.93) and CVD 0.63 (95% CI 0.42–0.93). When the general population was used as a reference group, the SRR for all-cause, cancer, and CVD mortality, as well as mortality for death from other causes were also significantly decreased, ranging from 0.36 (95% CI 0.29–0.44) for CVD and 0.68 (95% CI 0.58–0.80) for cancer. However, the SRR for death from external causes (1.25, 95% CI 1.03–1.52) and suicide (1.63, 95% CI 1.25–2.14) were significantly increased, indicating a higher rate of mortality. With the exception of death from external causes and suicide, all SRR of female physicians in reference to health professionals were higher than the respective SRR in reference to the general population.

We conducted a sensitivity analysis for the SRR of physicians in reference to the general population with increased observed values to compensate for the proportion of cases with missing causes of death. Assuming a percentage of cases with missing causes comparable to other occupational groups (upper limit 10.9%), marginally higher SRR for all causes of death were obtained, but overall conclusions did not change.

### Detailed causes of death for physicians in comparison to the general population

Table 3 provides a more detailed overview of specific causes of death for physicians, with data from the maximum observation period (1986–2020). SMR were calculated with the general population as a reference group. There were more than twice as many male (N=1230) than female physicians (N=471).

**Table 3:** Standardized mortality ratios (SMR) and 95% confidence intervals (CI) as well as observed (O) / expected (E) numbers of deaths in male and female physicians for detailed causes of death (1986–2020).

				ICD-10 ^a^		ICD-9 ^a^		Male physicians				Female physicians		
								O	E	SMR (95% CI)		O	E	SMR (95% CI)
All-cause mortality								1230	2787.7	0.44 (0.42–0.47) ^b^		471	722.2	0.65 (0.59–0.71) ^b^
	Infectious & parasitic disease			A00–B99		0010–1398		11	29.7	0.37 (0.19–0.66) ^b^		2	6.4	N/A ^c^
	Malignant neoplasms			C00–C97		1400–2089		395	878.5	0.45 (0.41–0.50) ^b^		239	342.9	0.70 (0.61–0.79) ^b^
		Head & neck		C00–C14, C30–32		1400–1499, 1600–1619		18	85.3	0.21 (0.13–0.33) ^b^		3	7.3	N/A ^c^
		Esophagus		C15		1500–1509		6	33.2	0.18 (0.07–0.39) ^b^		2	2.4	N/A ^c^
		Stomach		C16		1510–1519		19	49.6	0.38 (0.23–0.60) ^b^		5	15.2	N/A ^c^
		Colorectal		C18–C21		1530–1548		56	90.5	0.62 (0.47–0.80) ^b^		15	27.8	0.54 (0.30–0.89) ^b^
		Liver		C22		1550–1552		19	44.3	0.43 (0.26–0.67) ^b^		4	6.4	N/A ^c^
		Pancreas		C25		1570–1579		44	59.5	0.74 (0.54–0.99) ^b^		13	17.7	N/A ^c^
		Lung		C33–C34		1620–1629		65	253.2	0.26 (0.20–0.33) ^b^		31	51.2	0.61 (0.41–0.86) ^b^
		Skin		C43–C44		1720–1739		19	23.0	0.82 (0.50–1.29)		7	8.1	N/A ^c^
		Breast		C50		1740–1750						63	86.3	0.73 (0.56–0.93) ^b^
		Prostate		C61		1850		11	25.5	0.43 (0.21–0.77) ^b^				
		Brain & central nervous system		C70–C72		1910–1929		41	38.3	1.07 (0.77–1.45)		17	13.7	N/A ^c^
		Leukemia		C91–C95		2040–2089		17	25.0	0.68 (0.40–1.09)		7	9.6	N/A ^c^
		Other cancers						80	149.7	0.53 (0.42–0.67) ^b^		72	97.2	0.74 (0.58–0.93) ^b^
	Endocrine, nutritional & metabolic disorder			E00–E90		2430–2599		37	88.0	0.42 (0.30–0.58) ^b^		7	21.4	N/A ^c^
		Diabetes		E10–E14		2500–2509		25	52.8	0.47 (0.31–0.70) ^b^		3	11.0	N/A ^c^
	Mental and behavioral disorder			F01–F99		2900–3190		15	72.9	0.21 (0.12–0.34) ^b^		7	11.2	N/A ^c^
	Nervous system & sense organs disease			G00–H95		3200–3899		27	44.8	0.60 (0.40–0.88) ^b^		8	18.1	N/A ^c^
	Circulatory system disease			I00–I99		3900–4599		314	750.2	0.42 (0.37–0.47) ^b^		49	133.2	0.37 (0.27–0.49) ^b^
		Ischemic heart disease		I20–I25		4100–4149		193	440.4	0.44 (0.38–0.50) ^b^		15	50.2	0.30 (0.17–0.49) ^b^
			Myocardial infarction	I21–I22		4100–4109		136	312.8	0.43 (0.36–0.51) ^b^		14	33.4	0.42 (0.23–0.70) ^b^
		Cerebrovascular disease		I60–I69		4300–4380		49	105.2	0.47 (0.34–0.62) ^b^		14	35.2	0.40 (0.22–0.67) ^b^
		Other circulatory system disease		I00–I19, I26–I59, I70–I99		3900–4059, 4150–4299, 4400–4599		72	204.6	0.35 (0.28–0.44) ^b^		20	47.7	0.42 (0.26–0.65) ^b^
	Respiratory system disease			J00–J99		4600–5199		16	86.2	0.19 (0.11–0.30) ^b^		12	19.6	N/A ^c^
		Chronic lower respiratory disease		J40–J47		4900–4960		11	57.6	0.19 (0.10–0.34) ^b^		8	12.9	N/A ^c^
	Digestive system disease			K00–K92		5200–5799		69	282.0	0.24 (0.19–0.31) ^b^		18	51.2	0.35 (0.21–0.56) ^b^
		Chronic liver disease		K70, K73–K74		5710–5719		50	226.8	0.22 (0.16–0.29) ^b^		11	37.8	0.29 (0.15–0.52) ^b^
	External causes of death			V01–Y89		E8000–E9990		282	458.3	0.62 (0.55–0.69) ^b^		109	86.7	1.26 (1.03–1.52)
		Accidents		V01–X59, Y85–Y86		E8000–E9299		115	227.1	0.51 (0.42–0.61) ^b^		33	33.9	0.97 (0.67–1.37)
			Accidental poisoning ^d^	X40–X49		E8500–E8699		4	7.7	0.52 (0.14–1.33)		1	1.5	0.68 (0.02–3.78)
		Suicide		X60–X84, Y87.0		E9500–E9590		147	197.5	0.74 (0.63–0.87) ^b^		65	41.1	1.58 (1.22–2.02) ^b^
		Undetermined intent ^d^		Y10–Y34, Y87.2		E9800–E9890		16	18.3	0.87 (0.50–1.42)		6	5.4	1.10 (0.40–2.40)
Other causes of deaths								59	82.2	0.72 (0.55–0.93) ^b^		18	26.3	0.68 (0.41–1.08)

^a^ ICD = International Classification of Diseases. ^b^ Statistically significant at 5%. ^c^ Number of expected deaths lower than selection criterion, no SMR calculated. ^d^ Number of expected deaths lower than selection criterion, SMR calculated due to special interest

For male physicians, the SMR for all-cause mortality was 0.44 (95% CI 0.42–0.47), indicating that male physician mortality was less than half that of the general population. All other SMR were also <1, with the exception of cancer of the brain and central nervous system (1.07, 95% CI 0.77–1.45). There were several strikingly low SMR among the natural causes of death, such as 0.21 (95% CI 0.13–0.33) for head and neck cancer, 0.18 (95% CI 0.07–0.39) for esophageal cancer, 0.26 (95% CI 0.20–0.33) for lung cancer, 0.21 (95% CI 0.12–0.34) for mental and behavioral disorder, 0.19 for respiratory system disease (95% CI 0.11–0.30) and chronic lower respiratory disease (95% CI 0.10–0.34), 0.24 (95% CI 0.19–0.31) for digestive system disease and 0.22 (95% CI 0.16–0.29) for chronic liver disease. SMR tended to be closer to 1 for external causes of death like suicide (0.74, 95% CI 0.63–0.87), but mortality was still lower than in the general population, and SMR for categories like accidental poisoning (0.52, 95% CI 0.14–1.33) or deaths with undetermined intent (0.87, 95% CI 0.50–1.42) were not noticeably higher.

For female physicians, the SMR for all-cause mortality of 0.65 (95% CI 0.59–0.71) was higher than among males, but their mortality was still less than two thirds of the general population. All other SMR for deaths of natural causes were also <1, but overall fewer SMR were calculated due to the smaller sample size. The lowest SMR among female physicians were found in the section for CVD and ranged between 0.30 (95% CI 0.17–0.49) for ischemic heart disease and 0.42 (95% CI 0.26–0.65) for other circulatory system disease, in addition to 0.35 (95% CI 0.21–0.56) for digestive system disease and 0.29 (95% CI 0.15–0.52) for chronic liver disease. Mortality from external causes at 1.26 (95% CI 1.03–1.52) was significantly higher than in the general population, likely due to the higher rate of suicides (1.58, 95% CI 1.22–2.02). Again, the SMR for accidental poisoning and deaths with undetermined intent were not elevated.

## Discussion

This study found a considerably lower mortality among Austrian physicians compared to the general population, as well as a lower mortality compared to other selected professionals (data only available for males) and health professionals. Lower physician mortality than the general population is consistent with studies from the USA, Scandinavia and other European countries (10–15). Lower mortality compared to other (health) professionals is a more novel finding. The few existing studies have used different comparison groups to address the question of whether physicians stand out among other groups with similar SEP, with different findings. A study from the UK compared male physicians to the rather broad social class I and found their mortality to be lower (11), similar to an American study who used lawyers and other professionals (based on occupational census data) for comparison (12). A Finnish study also used professionals (predominantly engineers, managers and higher-education teachers) for comparison and found the physicians’ mortality to be higher (15). In a Norwegian study where physicians where compared to other university graduates, their mortality was reported as mostly similar (10).

The lower mortality among male and female physicians in the present study is mainly explained by significantly lower rates of cancer and CVD mortality. The analysis of more detailed causes of death confirms assumptions about a pattern of lower mortality for several causes of death with strong, evidence-based links to lifestyle-related health behaviors. This is particularly noticeable for smoking-associated causes of death, with low rates for lung cancer and respiratory disease. Overall, this is consistent with findings from other studies, although they either did not have an upper age limit (10, 12) or had a follow-up period beyond retirement age (11, 13–15). The present study also found lower mortality from breast cancer among female physicians, whereas other studies reported increased or similar rates compared to the general population (11, 13, 14). Since all these studies were conducted more than 20 years ago, this could be due to changes in breast cancer mortality for women with higher SEP. Recent data showed that their incidence is typically higher, but their case fatality rate lower when compared to women of lower SEP (4). We also did not find any evidence for comparably higher mortality from problematic alcohol use among physicians, while some older studies found mortality from chronic liver disease or liver cirrhosis that was at a similar level compared to the general population (11).

Similar to most other studies on physician mortality, we found an increased rate of suicide among female physicians compared to the general population, which is particularly striking among a long list of lower cause-specific mortality and was discussed in detail in a previous publication (33). There was, however, no indication of any “hidden” or misclassified suicides for both male and female physicians, which seemed to be the case in other studies (11, 12, 14).

With the exception of suicide among female physicians, the findings from this study support the notion that any potential occupational risk factors for physicians are compensated by their superior lifestyle, resulting in significantly lower mortality for a wide array of cause-specific mortality. The pattern of particularly low mortality for lifestyle-associated causes of death backs up the assumed pathway of better health behavior among physicians. The slightly lower mortality of physicians compared to other (health) professionals in Austria suggests beneficial factors beyond the expected extent of higher SEP. This could indicate a small positive influence of physicians’ extensive health and prevention knowledge, which is more extensive than in other health professions. A high level of health-related knowledge is likely to foster healthier lifestyle behaviors and more frequent use of preventive health services, leading to earlier detection of morbidity and improved treatment outcomes. There is some evidence that physicians display slightly, but not significantly better adherence to treatment instructions than non-physicians (39). Evidence on routine care and medical screening participation repeatedly showed that physicians often do not follow recommended guidelines when it comes to their own health, but some studies still found at least some preventive practices that were more common than among the general population (40, 41).

The strengths of this study are long observation periods that include recent data and the inclusion of several occupational groups on a nationwide level. The age range only includes working-age professionals, which results in a stronger focus on the period of active occupational influences (such as shift work and job stress) on mortality. To our knowledge, this is the first study to examine a variety of specific causes of death among Austrian physicians, and – due to a sufficiently high number of cases – we were able to analyze a comparably detailed selection of causes of death.

This study is also subject to limitations that should be taken into consideration. The lack of a sufficiently large number of women in two male-dominated professions (notaries and lawyers) meant that no comparison between female physicians and other professionals in general was possible. SRR and SMR based on the general population as a reference group are likely to be underestimated due to missing causes of death among physicians. Since this particular shortcoming applies to all occupational groups, comparisons between them are less affected.

### Concluding remarks

Our findings provide further support for earlier evidence on lower mortality among physicians that can be ascribed to lower mortality from lifestyle-associated causes of death. This study also found lower physician mortality in comparison to health professionals and other high-skilled professionals with similar SEP, confirming the importance of looking at the mortality outcomes of individual occupations. More research is needed on the causal pathways that influence occupational mortality risk among physicians and other health professionals, especially regarding increased suicide rates, which was the only significantly elevated cause of death among female physicians and should be subject to targeted prevention efforts. From a public health perspective, these findings also support the ongoing promotion of important health behaviors among physicians, underscoring the important role of physicians in leading their patients by example. Physicians with healthier habits not only live and practice longer, they are also more likely to provide adequate counselling and prevention advice to their patients (41, 42).
